# Caspase Mediated Synergistic Effect of *Boswellia serrata* Extract in Combination with Doxorubicin against Human Hepatocellular Carcinoma

**DOI:** 10.1155/2014/294143

**Published:** 2014-08-07

**Authors:** Mohammad Ahmed Khan, Mhaveer Singh, Masood Shah Khan, Abul Kalam Najmi, Sayeed Ahmad

**Affiliations:** ^1^Department of Pharmacology, Faculty of Pharmacy, Hamdard University, Hamdard Nagar, New Delhi 110062, India; ^2^Department of Pharmacognosy & Phytochemistry, Faculty of Pharmacy, Hamdard University (Jamia Hamdard), Hamdard Nagar, New Delhi 110062, India

## Abstract

The study investigated the growth-inhibiting and apoptosis mediating effects of *B. serrata* extract as monotherapy and combination therapy with DOX against hepatocellular carcinoma cell lines. Boswellic acid rich fraction of *B. serrata* extract was prepared. MTT assay on HepG2 and Hep3B cells was carried out using *B. serrata* alone and in combination with DOX. Further, caspase-3 activity, TNF-*α* level, and IL-6 level were estimated. Isobolographic analysis was carried out to evaluate the effect of combination therapy. Additionally, protective effect of *B. serrata* extract on DOX induced hepatic toxicity was also evaluated in Wistar rats. *B. serrata* extract inhibited growth of HepG2 (IC50 value of 21.21 ± 0.92 *μ*g/mL) as well as HepG2 (IC50 value of 18.65 ± 0.71 *μ*g/mL). DOX inhibited growth in HepG2 and Hep3B cells with an IC50 of 1.06 ± 0.04 *μ*g/mL and 1.92 ± 0.09 *μ*g/mL. Isobolographic analysis showed combination index (CI) of DOX and *B. serrata* extract of 0.53 ± 0.03 to 0.79 ± 0.02 suggesting synergistic behavior against the two cell lines. *B. serrata* extract also caused dose dependent increase in caspase-3 activity, TNF-*α* level, and IL-6 level which was higher (*P* < 0.001) with DOX (1 *μ*M) and *B. serrata* extract (20 *μ*g/mL) combination. *B. serrata* extract also protected Wistar rats against DOX induced hepatic toxicity.

## 1. Introduction

Hepatocellular carcinoma (HCC) is one of the most common cancers and the major leading cause of cancer related mortality globally [1]. The incidence of HCC is also increasing in Asia, Africa, Europe, and USA [2]. The lack of reliable biomarkers to detect HCC at early stage has caused the manifestation of advanced stages in most patients when surgical resection is not feasible [3]. Therefore, chemotherapy remains the viable strategy for treatment of inoperable cases of HCC. Doxorubicin (DOX) is one of the widely used anticancer drugs in the treatment of various malignancies including HCC [4]. However, the objective response rate with DOX as single agent is less than 20% whereas the median survival is only 4 months. Furthermore, clinical use of DOX is limited by severe adverse effects to nontumorous hepatic tissue [5, 6]. Impaired liver function, severe pancytopenia, and mucositis with DOX therapy can prompt to reduce the dosage [7]. However, the mechanisms of DOX-mediated cytotoxicity are different in cancer and normal tissues [8–10]. This difference in DOX-mediated toxicity can be used as an opportunity to improve the antitumor therapy with DOX.

Boswellia extracts, derived from the plant* Boswellia serrata* (*B. serrata*), have been known for their anti-inflammatory effects in the treatment of arthritis, ulcerative colitis, and Crohn disease [11]. Boswellic acids from* B. serrata* have also been proposed to provide antineoploastic activity through their antiproliferative and proapoptotic properties in multiple human cancer cell lines including meningioma cells [12], leukemia cells [13], melanoma cells, fibrosarcoma cells [14], colon cancer cells [15], and prostate cancer cells [16]. Boswellic acids (BA) from* Boswellia serrata* can induce apoptosis in cancer cells through activation of proapoptotic bcl-2 family and caspase-3 and upregulation of cell death receptors DR4 and TNF-R1 levels, leading to caspase-8 activation [17].

Multidrug therapy is a common practice in oncology since it can achieve better therapeutic outcome than monotherapy and can also minimize the adverse effects [18]. Thus it is imperative to design new combinatorial approaches with DOX that allow dose reduction, enhance the drug effectiveness, and reduce the toxicity.

The present study aimed to investigate the growth-inhibiting and apoptosis mediating effects of* B. serrata* extract alone as well as in combination with DOX in order to provide a new adjuvant therapy for HCC.

## 2. Materials and Methods

### 2.1. Procurement of Drug

Gum of* B. serrata* was procured from local market and samples were authenticated by Dr. H. B. Singh at NISCAIR (New Delhi), India.

### 2.2. Extraction and Isolation of Boswellic Acids

#### 2.2.1. Preparation of Methanolic Extract of* B. serrata*

Weighed quantity of* B. serrata* gum (50 g) was size-reduced and powdered gum was mixed with 100 mL methanol in a round bottom flask of 250 mL capacity and refluxed for 2 hours. The solvent was filtered to obtain methanolic extract (Part A) and process was repeated twice (parts B and C). All the three extracts, that is, parts A, B, and C, were mixed to obtain total methanolic extract. Methanolic extract was further concentrated on water bath and evaporated overnight. Residue obtained was weighed to obtain percentage yield.

#### 2.2.2. Isolation of Boswellic Acids from* B. serrata*

Concentrated methanolic extract of* B. serrata* was prepared as described. To the concentrated 20% Aq. KOH was added with constant stirring to dissolve till pH is 9-10. After the desired pH was obtained, the alkaline extract was filtered to remove any precipitate and further washed with equal volume of hexane thrice. After washing it was acidified with conc. HCl and final pH was adjusted to 2 so that formation of precipitate occurred. Precipitate so obtained was filtered and washed with water till neutral, dried in the air, and weighed.

### 2.3. HPLC Analysis

#### 2.3.1. Sample Preparation

*(a) Standard Stock Solution*. 50 mg of reference standard of boswellic acids was dissolved in 10 mL of HPLC grade methanol in volumetric flask. This solution sonicatied for 20 minutes.

*(b) Methanolic Extract of BS*. 1000 mg of methanolic extract of* B. serrata* was dissolved in 10 mL of HPLC grade methanol and solution was subjected to sonication for 20 minutes.

*(c) Isolated Boswellic Acids*. 50 mg of isolated boswellic acids were dissolved in 10 mL of HPLC grade methanol and solution was subjected to sonication for 20 minutes.

### 2.4. Quantification of Boswellic Acids in* B. serrata* Extracts

The analysis was carried out on a Waters Alliance e2695 separating module (Waters Co., MA, USA) using photo diode array detector (waters 2998) with autosampler and column oven. The instrument was controlled by use of Empower software installed with equipment for data collection and acquisition. Standard and sample solutions were filtered through 0.22 *μ*m syringe filter before injection and the separation was achieved by using a LiChroCART C_18_ column (25 × 4.6 mm, particle size 5 *μ*m). The mobile phase used was acetonitrile and 0.05% acetic acid in the ratio of 90 : 10 in gradient elution mode. Individual peaks were identified from retention time and concentrations were determined from the peak area for appropriate sample solutions using regression equation obtained from calibration plot. The detection of boswellic acids was done at the wavelength of 254 using PDA detector.

### 2.5. *In Vitro* Cytotoxicity Assay on HepG2 Cells

#### 2.5.1. Cell Culture Treatment

HepG2 and Hep3B cells were cultured in respective media supplemented with 100 U/mL penicillin, 100 U/mL streptomycin, and 10% foetal bovine serum (FBS) and kept at 37°C in a humidified atmosphere of 5% CO_2_. All cells were plated in cell culture flasks at least 24 h before treatment.

#### 2.5.2. Cell Viability Assay for* B. serrata* Extract* Per Se*

Cells were plated at an initial density of 8 × 10^3^ cells/well in a 96-well plate and treated with 10, 20, 40, 80, and 160 *μ*g/mL of* B. serrata* extract for 72 h. MTT solution was added to each well to a final concentration of 0.5 mg/mL. After 4 h of exposure at 37°C, the absorbance was measured at 570 nm using ELISA reader.

#### 2.5.3. Evaluation of* B. serrata* Extract with DOX

Cells were plated at an initial density of 8 × 10^3^ cells/well in a 96-well plate and treated with 1 *μ*M DOX* per se* and 1 *μ*M DOX in combination with 20 *μ*g/mL of* B. serrata* extract for 72 h. MTT solution was added to each well to a final concentration of 0.5 mg/mL. After 4 h of exposure at 37°C the absorbance was measured at 570 nm using ELISA reader.

### 2.6. Cytokine Assay

Cells were collected after treatment and lysed with 50 *μ*L cell lysis buffer. Then the cell lysate was centrifuged and the supernatants were retained. TNF-*α* quantification, caspase-3 activity, and IL-6 level estimation were carried out using commercially available ELISA kits. NF-*κ*B expression was evaluated using western blotting.

### 2.7. Isobolographic Analysis

To study the interaction between DOX and* B. serrata* extract, Cells were treated with various dilutions of DOX in the presence or absence of* B. serrata* extract at 10, 20, and 30 *μ*g/mL and IC 50 values were determined by plotting the percentage of cell survival as a function of drug concentration [18, 19]. The interactions between DOX and* B. Serrata* extract were evaluated by isobolographic analysis, a dose-oriented geometric method of assessing drug interactions [20]. For 50 percent toxicity, the combination index (CI) values were calculated based on the equation stated below:

(1) Combination  Index  (CI)=D1Dx1+D2Dx2+α{(D1∗D2)(Dx1∗Dx2)},

where *Dx*1 is dose of drug 1 to produce 50 percent cell kill alone; *D*1 is dose of drug 1 to produce 50 percent cell kill in combination with *D*2; *Dx*2 is dose of drug 2 to produce 50 percent cell kill alone; *D*2 is dose of drug 2 to produce 50 percent cell kill in combination with *D*1; and *α* = 1.

### 2.8. Animals and Treatment Protocols

Adult albino (Wistar strain) rats weighing between 150 and 200 gm were used for the study. The animals were procured from Central Animal House Facility, Jamia Hamdard. Throughout the experimental period, the animals were housed in cages under room temperature (20 ± 2°C) and relative humidity (60–70%) and exposed to 12 : 12 h light  : dark cycle. They were fed with standard laboratory diet supplied by M/S Ashirwad feed industry and water* ad libitum*.

The study was approved by Institutional Animal Ethics Committee of the University and animals received humane treatment as per Committee for the Purpose of Control and Supervision on Experiments on Animals (CPCSEA) guidelines. Animals were divided into five treatment groups. Group I was control and received normal saline 1 mL/kg p.o. for 30 days. Group II (toxic control) received normal saline 1 mL/kg p.o. for 30 days with DOX 30 mg/kg i.p. on 28th day. Group III (DOX + BS250) and IV (DOX + BS500) were given* B. serrata* extract at 250 and 500 mg/kg p.o., respectively, for 30 days with DOX 30 mg/kg i.p. single dose on 28th day. Group V (DOX + SY100) served as standard and was administered silymarin 100 mg/kg for p.o. for 30 days with DOX 30 mg/kg i.p. on 28th day.

### 2.9. Doxorubicin Hepatic Toxicity Assay

72 hours after DOX administration, blood was withdrawn from the tail vein for serum estimations. Followed by this animals were sacrificed under high dose of anaesthesia and liver tissue was excised out washed with ice cold saline and stored for biochemical and histopathological studies.

*Biochemical Estimations in Serum and Tissue.* Serum samples were used for liver function test and estimation of serum SGOT, SGPT, albumin, bilirubin, and alkaline phosphatase levels using commercially available kits. Extent of lipid peroxidation was measured as by thiobarbituric acid (TBA) reaction with malondialdehyde (MDA) according to the method described by [21].

### 2.10. Histopathology

The excised liver tissues were fixed in 10% neutral buffered formalin and sections of maximum 5 *μ*m thickness were cut. Further, sections were stained with hematoxylin and eosin (H&E) for histopathology. The histomorphological evaluation of the liver sections was done by a pathologist unacquainted with the treatment groups.

### 2.11. Statistical Analysis

The Data was represented as mean ± standard deviation (SD). One-way analysis of variance (ANOVA) was used to compare the means of all groups. The Tukey-Kramer post hoc test was used to test the significance among groups. The value of *P* < 0.05 was considered to be statistically significant. GraphPad Prism was used for statistical analysis.

## 3. Results

### 3.1. HPLC Analysis of Boswellic Acids

**Figure 1:**
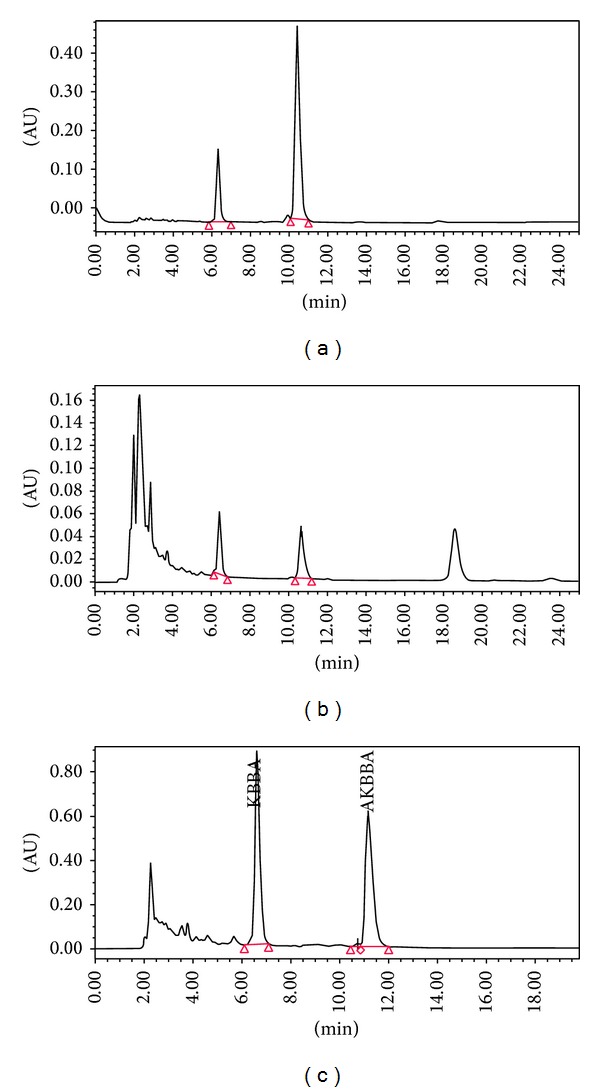
HPLC chromatogram of (a) standard solution showing KBBA and AKBBA; (b) methanolic extract showing KBBA and AKBBA; and (c) isolated boswellic acid extract showing KBBA and AKBBA at 254 nm.

The HPLC-PDA method was developed and successfully employed for the identification and quantification of* acetyl-keto-*β*-boswellic acid* (AKBA) and* keto-*β*-boswellic acid* (KBA) in prepared extracts of* B. serrata*. Identification of the peaks in the sample chromatograms were carried out by comparing retention and PDA spectra of each component (Figures 1(a)–1(c)). The contents AKBA and KBA were calculated from the corresponding calibration curve. The quantities of AKBA and KBA were 5% and 1.87% w/w, respectively, in prepared extract whereas the content of these two components were 0.917% and 0.71% w/w, respectively, in methanolic extracts.

### 3.2. *In Vitro* Cytotoxicity Assay

#### 3.2.1. Antiproliferative Effect of* B. serrata* Extract

**Figure 2:**
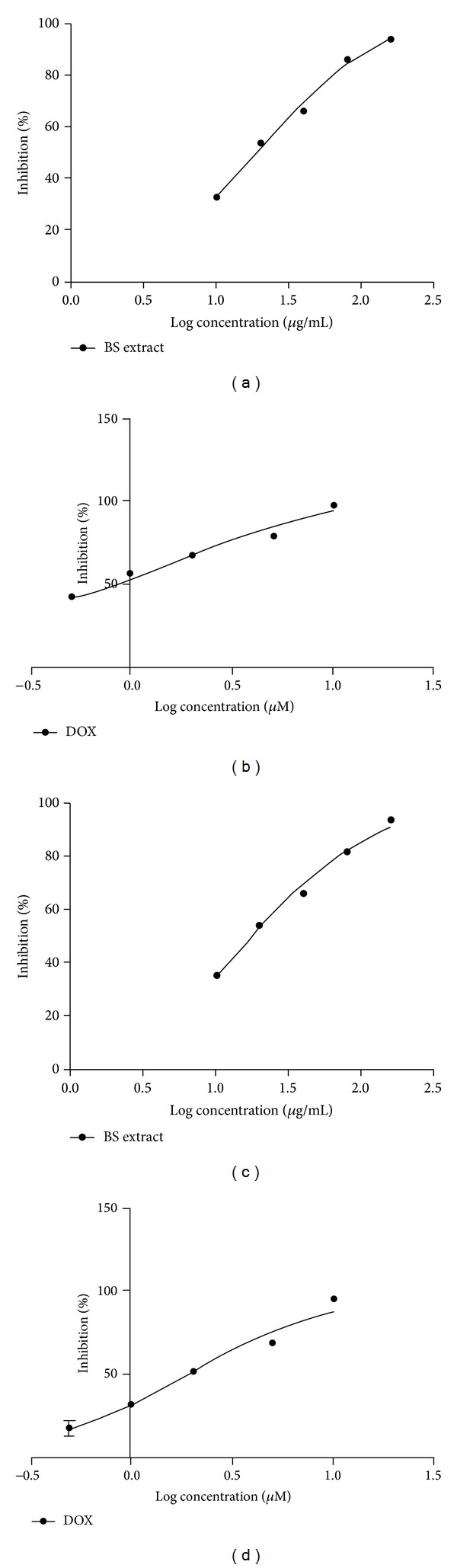
Concentration response curve for MTT assay of (a)* Boswellia serrata* (BS) extract and (b) doxorubicin (DOX) against HepG2 cells and (c)* Boswellia serrata* (BS) extract and (d) doxorubicin (DOX) against Hep3B cells.

*B. serrata* extract inhibited proliferation of HepG2 and Hep3B cells with an IC50 value of 21.21 ± 0.92 *μ*g/mL and 18.65 ± 0.71 *μ*g/mL, respectively. DOX showed IC50 of 1.06 ± 0.04 *μ*g/mL and 1.92 ± 0.09 against HepG2 and Hep3B cells, respectively (Figures 2(a)–2(d)).

#### 3.2.2. Effect of* B. serrata* Extract in Combination with DOX

The combined effects of DOX and* B. serrata* extract on cell proliferation were evaluated by isobolographic analysis method. The CI values ranged from 0.53 ± 0.03 to 0.76 ± 0.04 for 50 percent cell kill suggesting synergistic behavior between* B. serrata* extract and DOX against both HepG2 cells. The CI value in Hep3B cells ranged from 0.55 ± 0.04 to 0.79 ± 0.02 (Table 1).

### 3.3. Cytokine Levels

#### 3.3.1. Caspase-3 Activity

**Figure 3:**
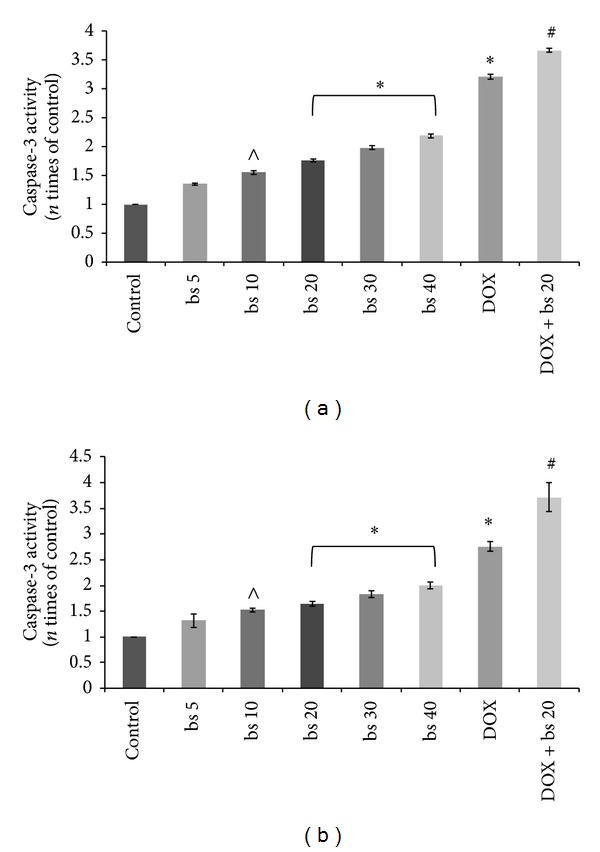
Effect of different treatment groups on Caspase-3 activity in HepG2 cells. (bs 5-Boswellia extract 5 *μ*g/mL; bs 10-Boswellia extract 10 *μ*g/mL; bs 20-Boswellia extract 20 *μ*g/mL; bs 30-Boswellia extract 30 *μ*g/mL; bs 40-Boswellia extract 40 *μ*g/mL; DOX: doxorubicin 1 *μ*M; DOX + bs 20: doxorubicin 1 *μ*M + 20-Boswellia extract 20 *μ*g/mL. ^∧^ *P* < 0.05 versus control;*P < 0.001 versus control; ^#^ *P* < 0.001 versus DOX); (a) HepG2 cells and (b) Hep3B cells.

Effect of* B. serrata* extract* per se* and in combination with DOX on capsase-3 activity is shown in Figure 3.* B. serrata* extract treatments at 5 *μ*g/mL showed insignificant difference compared to control treatment. Treatment at 10 *μ*g/mL increased caspase activity significantly (*P* < 0.01) to almost 1.5 fold of control level. However, treatment at 20–40 *μ*g/mL increased caspase-3 activity to even higher level (*P* < 0.001). Results showed that* B. serrata* alone and in combination with DOX showed significant (*P* < 0.001) increased expression of caspase-3 activity compared to control. The expression of caspase-3 in combination treatment was significantly (*P* < 0.001) different from single agent treatment.

#### 3.3.2. TNF-*α* Level

**Figure 4:**
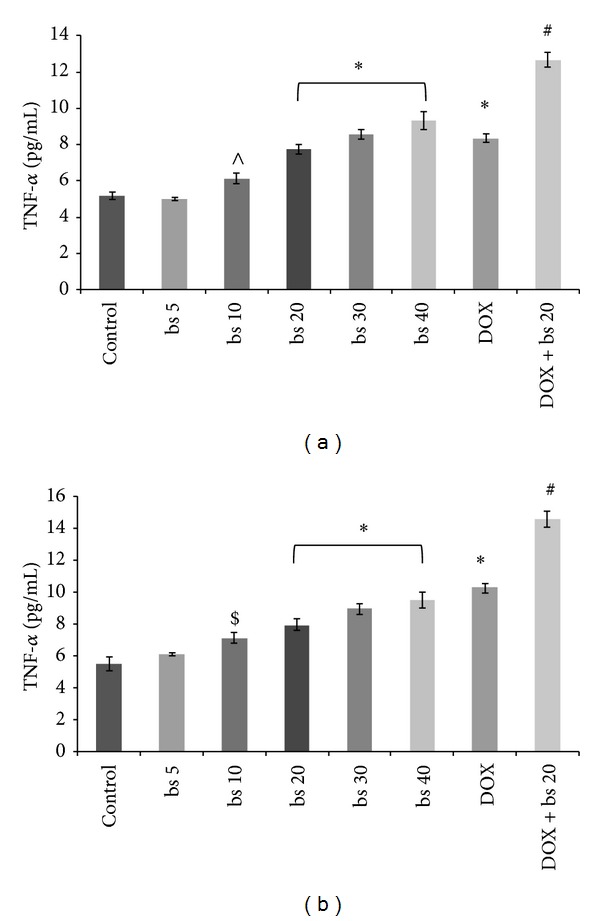
Effect of different treatment groups on TNF-*α* levels in HepG2 cells. (bs 5-Boswellia extract 5 *μ*g/mL; bs 10-Boswellia extract 10 *μ*g/mL; bs 20-Boswellia extract 20 *μ*g/mL; bs 30-Boswellia extract 30 *μ*g/mL; bs 40-Boswellia extract 40 *μ*g/mL; DOX: doxorubicin 1 *μ*M; DOX + bs 20: doxorubicin 1 *μ*M+ bs 20-Boswellia extract 20 *μ*g/mL. ^∧^ *P* < 0.05 versus control; *P < 0.001 versus control; ^#^ *P* < 0.001 versus DOX.); (a) HepG2 cells and (b) Hep3B cells.

*B. serrata* extract treatments significantly increased TNF-*α* level compared to control (Figure 4) except at the dose of 5 *μ*g/mL (*P* > 0.05). TNF-*α* level at the doses of 20–40 *μ*g/mL was significantly higher (*P* < 0.001 versus control) than 10 *μ*g/mL in both HepG2 cells (*P* < 0.01 versus control, Figure 4(a)) as well as in Hep3B cells (*P* < 0.05 versus control, Figure 4(b)). Further, results showed that treatment with DOX alone and in combination with* B. serrata* extract significantly (*P* < 0.001) enhanced TNF-*α* level compared to control. TNF-*α* level in combination treatment was significantly (*P* < 0.001) higher from treatment with* B. serrata* extract or DOX.

#### 3.3.3. IL-6 Level

**Figure 5:**
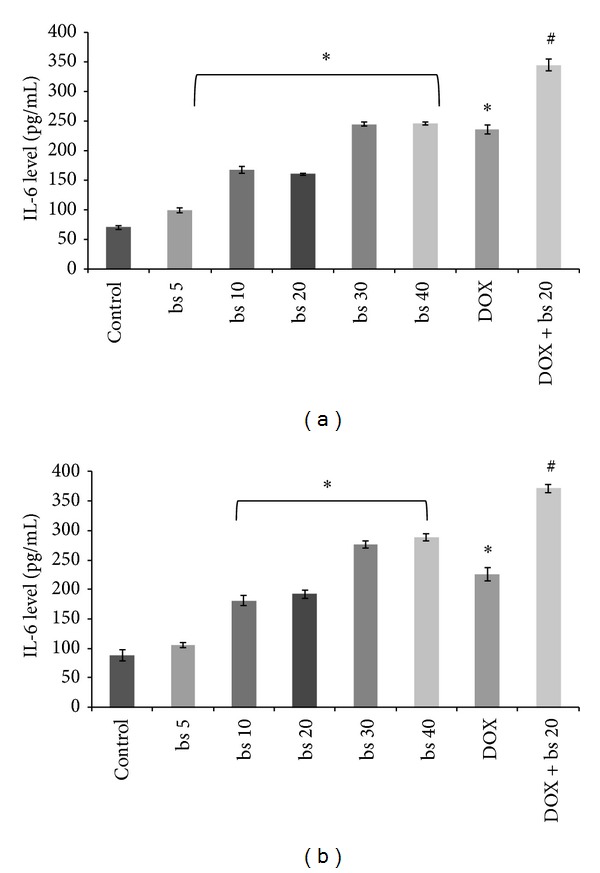
Effect of different treatment groups on IL-6 levels in HepG2 cells. (bs 5-Boswellia extract 5 *μ*g/mL; bs 10-Boswellia extract 10 *μ*g/mL; bs 20-Boswellia extract 20 *μ*g/mL; bs 30-Boswellia extract 30 *μ*g/mL; bs 40-Boswellia extract 40 *μ*g/mL; DOX: doxorubicin 1 *μ*M; DOX + bs 20: doxorubicin 1 *μ*M+ bs 20-Boswellia extract 20 *μ*g/mL.*P < 0.001 versus control; ^#^ *P* < 0.001 versus DOX.); (a) HepG2 cells and(b) Hep3B cells.

IL-6 level in HepG2 treated with* B. serrata* extract showed dose dependent significant (*P* < 0.001) increase compared to control (Figure 5(a)). In Hep3B cells treatment showed insignificant change at a dose of 5 *μ*g/mL and dose dependent increase in IL-6 level at doses above 5 *μ*g/mL. DOX and combination treatment of DOX and* B. serrata* extract also caused increased IL-6 levels (*P* < 0.001 versus control). The expression of IL-6 in combination treatment was significantly (*P* < 0.001) different from any single drug treatment.

#### 3.3.4. NF-*κ*B Expression

**Figure 6:**
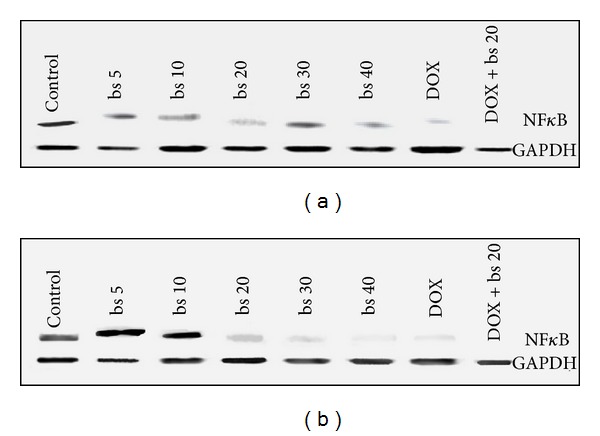
Western blotting to determine expressions of NF-*κ*b; (a) HepG2 cells and (b) Hep3B cells.

*B. serrata* extract showed dose dependent reduction in antiapoptotic protein NF-*κ*B expression in HepG2 cells and combination treatment with doxorubicin also decreased expression of NF-*κ*B (Figure 6(a)). Comparatively, the expression was less in combination than single treatments. Similar results were obtained in Hep3B cells (Figure 6(b)).

### 3.4. Hepatic Toxicity Assay

#### 3.4.1. Hepatic Enzyme Activity

The activity of serum markers of liver injury SGOT, SGPT, and ALP was significantly elevated (*P* < 0.001) in rats treated with DOX due to toxic effect (Table 2). Although the enzyme activities were significantly lower in the control group. Treatment with* B. serrata* extract both at 250 and 500 mg/kg/day significantly reduced (*P* < 0.001) activities of SGOT, SGPT, and ALP in comparison to DOX treated group. In the present study, enzyme levels in the standard group treated with sylimarin (100 mg/kg/day) showed comparable improvement (*P* > 0.05) to groups treated with* B. serrata* extract.

#### 3.4.2. Serum Protein Levels

The serum albumin levels in toxicant group were significantly reduced (*P* < 0.001) compared to control animals (Table 2). Treatment with* B. serrata* extract restored albumin levels to significantly higher level compared to DOX treated animals (*P* < 0.001); this effect was also comparable to standard group (*P* > 0.05). Serum bilirubin level was significantly increased in DOX treated animals (*P* < 0.001). All the treatment significantly restored bilirubin level (*P* < 0.001 versus control). Within the liver tissues of DOX treated animals, there was significant alteration detected in the MDA levels compared to the control group, which was >6 fold of control level. A substantial decrease was observed in the MDA levels of the rats, which were administered* B. serrata* extract at 250 and 500 mg/kg/day (*P* < 0.05).

#### 3.4.3. Histopathology

**Figure 7:**
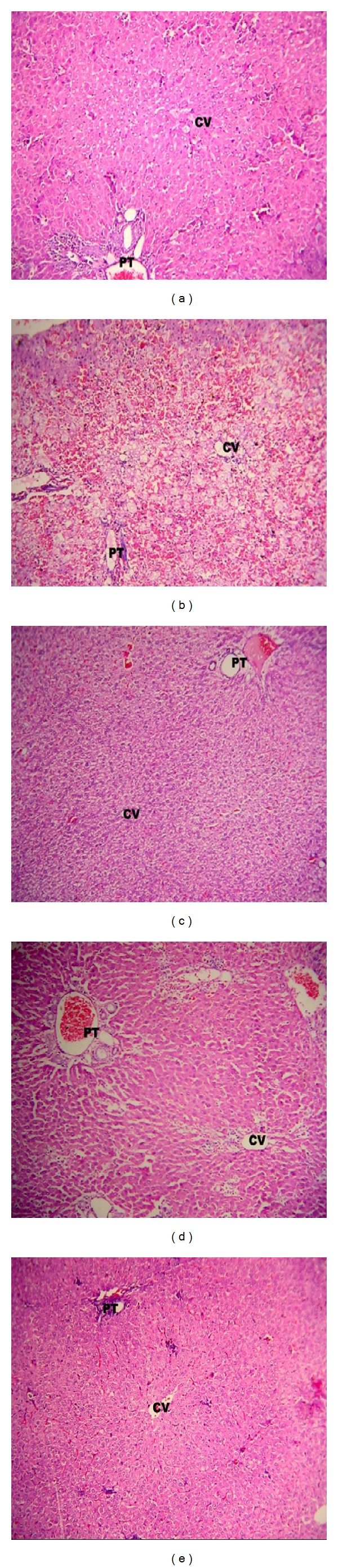
Showing liver section prepared from tissues of different treatment groups and stained by H&E staining. (a) Control group showing normal polygonal cells with prominent round nuclei and well preserved cytoplasm and visible central vein; (b) DOX intoxicated group showing massive fatty changes, necrosis, ballooning degeneration and broad infiltration of lymphocytes, loose cellular boundaries, vacuolated hepatocytes, and mild periportal inflammatory cell infiltration; (c) group treated with* B. serrata* extract at 250 mg/kg showing more or less normal lobular pattern with mild degree of fatty changes; (d) group treated with* B. serrata* extract at 500 mg/kg showing centrizonal area with almost nil inflammatory cell infiltration; and (e) standard group showing almost normal hepatic architecture.

The histopathological examination showed that the control group tissues have normal polygonal cells with prominent round nuclei and well preserved cytoplasm, prominent nucleus, nucleolus, and visible central vein (Figure 7(a)) (normal hepatic architecture). The liver sections of the animal intoxicated with DOX showed massive fatty changes, necrosis, ballooning degeneration and broad infiltration of lymphocytes, loose cellular boundaries, vacuolated hepatocytes, and mild periportal inflammatory cell infiltration (Figure 7(b)). The histological architecture of liver sections of animal treated with* B. serrata* extract at 250 mg/kg showed more or less normal lobular pattern with mild degree of fatty changes (Figure 7(c)). The animal treated with* B. serrata* extract at 500 mg/kg showed the centrizonal area with almost nil inflammatory cell infiltration (Figure 7(d)). The results are comparable with standard (Figure 7(e)) and control.

## 4. Discussion

HCC is one of the most lethal forms of cancers. This is substantiated by the facts that even though there are significant advances in surgery and chemotherapy, the majority of patients with HCC die within one year of diagnosis [22]. Additionally, the current treatment approaches are associated with side effects leading to inadequate outcomes. Thus, there is desperate need for new treatment options of HCC. Cancer therapy with single drug confers quick development of drug resistance where combination therapy simultaneously with two or more drugs has ability to improve results and reduce the chances of resistance [23]. Although there have been advancement in drug targeting as well as combination therapy, no curative treatment options are yet achieved. This problem requires development of better therapeutic agents and combination strategies [24, 25]. Recent studies on brain tumors, leukemic, and colon cancer cells indicate that boswellic acids from* B. serrata* may have antiproliferative effects [15]. DOX is one of the most commonly used anticancer agents against HCC; however, it is associated with severe toxicities to vital organs [19, 20, 26]. In the present study, we demonstrated that* B. serrata* extract containing boswellic acids in combination with DOX was effective in a synergistic manner in inhibition of tumor growth of HCC* in vitro* and also protected against toxicity* in vivo*. Our results indicate that the anticancer activity of* B. serrata* alone and in combination with DOX was mediated via activation of caspase cascade and induction of apoptosis.

To our knowledge, this is the first study that demonstrates effectiveness of* B. serrata* in combination with DOX against HCC. In this study, we demonstrated that* B. serrata* inhibits HepG2 cells proliferation* in vitro* with IC50 value of 21.21 ± 0.92 *μ*g/mL (Figure 2(a)) and Hep3B cells with 18.65.21 ± 0.71 *μ*g/mL (Figure 2(b)). This was lower than the IC50 observed with AKBA treatment in other cancer cells [16, 27, 28].

The antiproliferative potential of different agents has been found to vary based on sensitivity and type of cancer. In our study, HepG2 and Hep3B cells were used to establish interaction between* B. serrata* extract and DOX using isobolographic analysis. Isobolographic analysis has been commonly used to assess the interaction between two antitumor agents. It also provides qualitative as well as quantitative measure of nature and extent of interaction [29]. In the present investigation, isobolographic analysis showed that* B. serrata* enhanced the cytotoxicity of DOX in both HepG2 cells (CI value 0.53–0.76) Hep3B cells (CI value 0.55–0.79) in a synergistic manner (Table 1).

Inhibition of cell proliferation by induction of apoptotsis is one of the important mechanisms by which anticancer agents act [30]. Ottewel et al. [2008] have previously shown that combination of zoledronic acid and DOX caused significant (*P* < 0.05) increase in caspase-3 positive cells in MDA-G8 breast tumor xenografts compared to single treatment. To study the possible mechanism involved in the anticancer activity of* B. serrata* extract and combination, we evaluated induction of apoptosis of HepG2 cells by measuring caspase-3 activity. Induction of caspase-3 has been demonstrated following boswellic acid treatment in colon cancer [15], lukemic cells [31], and prostate cancer cells [32]. Induction of apoptosis and expression of cleaved caspase 3 was significantly (*P* < 0.001) induced* in vitro* by combination treatment compared to* B. serrata* or DOX alone (Figure 3). In* in vitro* increase in caspase-3 activity correlated very well with cytotoxicity thus confirming that caspase mediated apoptosis is an important pathway associated with the anticancer activity of these agents. Further, TNF-*α* are cytokines which can stimulate the acute inflammatory response. TNF-*α* is also able to induce apoptotis and inhibit tumorigenesis via caspase-3 [33–36]. In this study, the secretion of TNF-*α* increased in a dose dependent manner in both HepG2 and Hep3B cells (Figure 4). As observed with caspase-3, TNF-*α* level with combination treatment was significantly higher (*P* < 0.001) than single use of* B. serrata* or DOX. These results reinforced that apoptosis of HCC cells as induced by TNF-*α* treatment is mediated through caspase-3. The tumor regression by DOX and combination was also mediated through increased expression of IL-6 levels (Figure 5). DOX is reported to increase IL-6 expression through generation of oxidative stress.* B. serrata* is a proven anti-inflammatory agent in normal tissue, but our study showed that it can* per se* increase IL-6 level in carcinoma cells. This effect was synergistic in combination with DOX. To gain more insights on the mechanisms of increased IL-6 level with boswellic and combination therapy, other nonapoptotic signaling pathways need to be investigated. NF-*κ*B has been shown to promote cell survival signals leading to inhibition of apoptosis and cancer growth [37]. Previously boswellic acid has been shown to inhibit TNF-*α* induced activation of NF-*κ*B [38] and in our experiment* B. serrata* extract and combination treatment inhibited NF-*κ*B activation (Figure 6). This confirmed that combination of* B. serrata* extract and doxorubicin exhibit synergistic apoptosis mediated via NF-*κ*B.

After establishing the efficacy of* B. serrata* extract alone and combination with DOX on HepG2 cells* in vitro*, we designed* in vivo* experiments to test the efficacy of* B. serrata* extract against DOX-induced hepatic toxicity in Wistar rats. Previous studies have shown that prophylactic oral administration of* B. serrata* extracts prevented the development and progression of hepatic fibrosis mice [39]. Therefore, we evaluated* B. serrata* extract for* in vivo* hepatoprotective efficacy at two different dose levels of 250 to 500 mg/kg/day against DOX. Our results demonstrated that oral administration of* B. serrata* extract at 250 and 500 mg/kg/day showed significant improvement in liver function (Table 2). Animals were treated with DOX at 30 mg/kg (i.p.), a dose that causes acute toxicity [40]. The acute hepatotoxicity of DOX was clearly revealed by the increase in serum biochemical markers SGOT, SGPT, ALP, and bilirubin and decrease in serum albumin level. The most important cause of DOX toxicity is oxidative stress [41]. DOX generates free radical either by enzymatic formation of semiquinone by NADH dependent reductase or by a nonenzymatic reaction with iron. Further, DOX also decreases liver tissues ability to detoxify reactive oxygen species [42]. In the present study, generation of oxidative stress by one of these mechanisms should have contributed to hepatic dysfunction. However, the cotreatment of DOX and* B. serrata* extract resulted in a partial reversal of DOX-induced changes in these biomarkers (*P* < 0.001). Acute histopathological changes in DOX-treated livers were also reversed by* B. serrata* extract administration.* B. serrata* gum is a clinically proven anti-inflammatory agent. Boswellic acids from gum have been successfully used to prevent injury responses [32] and are effective agents in preventing oxidant-induced injury responses [43]. Boswellic acids can scavenge a broad spectrum of reactive oxygen and nitrogen species [44]. Recently the extract of* B. serrata* has been shown to possess active antioxidant substances which can exert protective effects against acute oxidative stress [45]. This scenario is suggestive of* B. serrata* extracts hepatoprotective nature through reduction of DOX-mediated oxidative stress. To further confirm this mechanism, Malondialdehyde (MDA), a product of lipid peroxidation, was measured in hepatic tissues of the treatment groups to evaluate the DOX-induced damage caused by oxidative stress [46]. MDA levels were significantly increased (*P* < 0.001) from control level showing DOX-induced lipid peroxidation. Results showed that treatment with* B. serrata* extract at 250 and 500 mg/kg significantly (*P* < 0.001) prevented DOX-induced lipid peroxidation and oxidative damage thus confirming our results.

## 5. Conclusion

In conclusion, our data provides convincing evidence that combination treatment is effective against HepG2 and Hep3B cells by induction of apoptosis. While the currently available chemotherapeutic options are associated with unavoidable adverse effects, oral* B. serrata* extract provides hope as a useful anticancer agent with significantly lower toxicity on normal liver tissue. Thus the use of synergistically acting* B. serrata* extracts and DOX combination therapy could be a novel strategy for the treatment for HCC and probably will have lesser toxicity compared to currently used regimens.

## Figures and Tables

**Table 1:** Result of Isobolographic analysis showing combination index (CI).

Treatment	CI	
	HepG2	Hep3B
DOX + bs10	0.76	0.79
DOX + bs20	0.54	0.56
DOX + bs30	0.53	0.55

The CI values represent mean of four experiments. CI.1.3: antagonism; CI 1.1–1.3: moderate antagonism; CI 0.9–1.1: additive effect; CI 0.8–0.9: slight synergism; CI 0.6–0.8: moderate synergism; CI 0.4–0.6: synergism; and CI 0.2–0.4: strong synergism. DOX + bs10-doxorubicin 1 *μ*M + Boswellia extract 10 *μ*g/mL; DOX + bs20-doxorubicin 1 *μ*M + Boswellia extract 20 *μ*g/mL; and DOX + bs30-doxorubicin 1 *μ*M + Boswellia extract 30 *μ*g/mL.

**Table 2:** Effect of Boswellia extract on various hepatic biomarkers against doxorubicin induced hepatic toxicity.

	Control	DOX	DOX + BS250	DOX + BS500	DOX + SY100
SGPT (IU/L)	83.44 ± 13.01	360.05 ± 17.9*	171.56 ± 12.7^b,a^	169.69 ± 10.2^b,a^	135.94 ± 8.1^b^
SGOT (IU/L)	57.50 ± 7.72	209.17 ± 9.04*	113.01 ± 8.33^b^	106.57 ± 8.94^b^	110.03 ± 9.37^b^
Albumin (mg/dL)	4.54 ± 0.23	1.70 ± 0.32*	3.35 ± 0.26^b^	3.43 ± 0.17^b^	3.38 ± 0.11^b^
Alkaline Phosphatase (IU/L)	136.68 ± 12.3	414.55 ± 15.7*	158.98 ± 12.8^b^	155.18 ± 11.1^b^	157.35 ± 13.4^b^
Total Bilirubin (mg/dL)	1.63 ± 0.22	6.68 ± 0.30*	4.16 ± 0.22^b,z^	3.79 ± 0.14^z^	2.95 ± 0.22^b^
MDA (nmoles/mg)	57.51 ± 5.59	332.63 ± 13.03*	90.73 ± 4.62^b,z^	66.81 ± 3.29^b^	64.14 ± 2.43^b^

*P < 0.001 versus control; ^b^ *P* < 0.01 versus DOX; ^z^ *P* < 0.001 versus DOX + SY100; and ^a^ *P* < 0.01 versus DOX + SY100.

## References

[1] Farazi PA, DePinho RA (2006). Hepatocellular carcinoma pathogenesis: from genes to environment. *Nature Reviews Cancer*.

[2] Hossain MA, Kim DH, Jang JY (2012). Aspirin enhances doxorubicin-induced apoptosis and reduces tumor growth in human hepatocellular carcinoma cells in vitro and in vivo. *International Journal of Oncology*.

[3] Nowak AK, Chow PKH, Findlay M (2004). Systemic therapy for advanced hepatocellular carcinoma: a review. *European Journal of Cancer*.

[4] Dudeck O, Ricke J (2011). Advances in regional chemotherapy of the liver. *Expert Opinion on Drug Delivery*.

[5] Silber JH, Barber G, Paz-Ares L, Dobbs N, Twelves C (1995). Doxorubicin-induced cardiotoxicity. *The New England Journal of Medicine*.

[6] King PD, Perry MC (2001). Hepatotoxicity of chemotherapy. *Oncologist*.

[7] Benjamin RS, Wiernik PH, Bachur NR (1974). Adriamycin chemotherapy: efficacy, safety, and pharmacologic basis of an intermittent single high dosage schedule. *Cancer*.

[8] Wang S, Konorev EA, Kotamraju S, Joseph J, Kalivendi S, Kalyanaraman B (2004). Doxorubicin induces apoptosis in normal and tumor cells via distinctly different mechanisms: intermediacy of H_2_O_2_-and _p53_-dependent pathways. *Journal of Biological Chemistry*.

[9] Hovorka O, Subr V, Větvička D (2010). Spectral analysis of doxorubicin accumulation and the indirect quantification of its DNA intercalation. *European Journal of Pharmaceutics and Biopharmaceutics*.

[10] Mukhopadhyay P, Rajesh M, Bátkai S (2009). Role of superoxide, nitric oxide, and peroxynitrite in doxorubicin-induced cell death in vivo and in vitro. *The American Journal of Physiology—Heart and Circulatory Physiology*.

[11] Shen Y, Takahashi M, Byun H (2012). Boswellic acid induces epigenetic alterations by modulating DNA methylation in colorectal cancer cells. *Cancer Biology and Therapy*.

[12] Yong SP, Lee JH, Bondar J, Harwalkar JA, Safayhi H, Golubic M (2002). Cytotoxic action of acetyl-11-keto-*β*-boswellic acid (AKBA) on meningioma cells. *Planta Medica*.

[13] Shao Y, Ho C, Chin C, Badmaev V, Ma W, Huang M (1998). Inhibitory activity of boswellic acids from *Boswellia serrata* against human leukemia HL-60 cells in culture. *Planta Medica*.

[14] Zhao L, Wientjes MG, Au JL- (2004). Evaluation of combination chemotherapy: integration of nonlinear regression, curve shift, isobologram, and combination index analyses. *Clinical Cancer Research*.

[15] Liu JJ, Nilsson Å, Oredsson S, Badmaev V, Zhao WZ, Duan RD (2002). Boswellic acids trigger apoptosis via a pathway dependent on caspase-8 activation but independent on Fas/Fas ligand interaction in colon cancer HT-29 cells. *Carcinogenesis*.

[16] Pang X, Yi Z, Zhang X (2009). Acetyl-11-keto-*β*-boswellic acid inhibits prostate tumor growth by suppressing vascular endothelial growth factor receptor 2-mediated angiogenesis. *Cancer Research*.

[17] Bhushan S, Kumar A, Malik F (2007). A triterpenediol from *Boswellia serrata* induces apoptosis through both the intrinsic and extrinsic apoptotic pathways in human leukemia HL-60 cells. *Apoptosis*.

[18] Kirk R (2013). Targeted therapies: the maths behind combination therapy. *Nature Reviews Clinical Oncology*.

[19] Yeo W, Mok TS, Zee B (2005). A randomized phase III study of doxorubicin versus cisplatin/interferon *α*-2b/doxorubicin/fluorouracil (PIAF) combination chemotherapy for unresectable hepatocellular carcinoma. *Journal of the National Cancer Institute*.

[20] Forner A, Llovet JM, Bruix J (2012). Hepatocellular carcinoma. *The Lancet*.

[21] Iqbal M, Dubey K, Anwer T, Ashish A, Pillai KK (2008). Protective effects of telmisartan against acute doxorubicin-induced cardiotoxicity in rats. *Pharmacological Reports*.

[22] Khan M, Li T, Ahmad Khan MK (2013). Alantolactone induces apoptosis in HepG2 cells through GSH depletion, inhibition of STAT3 activation, and mitochondrial dysfunction. *BioMed Research International*.

[23] Komarova NL, Boland CR (2013). Cancer: calculated treatment. *Nature*.

[24] Yang Y, Yan X, Duan W (2013). Pterostilbene exerts antitumor activity via the Notch1 signaling pathway in human lung adenocarcinoma cells. *PLoS ONE*.

[25] Liu Y, Wang L, Wu Y (2013). Pterostilbene exerts antitumor activity against human osteosarcoma cells by inhibiting the JAK2/STAT3 signaling pathway. *Toxicology*.

[26] Sharma P, Saini SD, Kuhn LB (2011). Knowledge of hepatocellular carcinoma screening guidelines and clinical practices among gastroenterologists. *Digestive Diseases and Sciences*.

[27] Hoernlein RF, Orlikowsky T, Zehrer C (1999). Acetyl-11-keto-*β*-boswellic acid induces apoptosis in HL-60 and CCRF- CEM cells and inhibits topoisomerase I. *Journal of Pharmacology and Experimental Therapeutics*.

[28] Lu M, Xia L, Hua H, Jing Y (2008). Acetyl-keto-*β*-boswellic acid induces apoptosis through a death receptor 5-mediated pathway in prostate cancer cells. *Cancer Research*.

[29] Zhao L, Wientjes MG, Au JL (2004). Evaluation of combination chemotherapy: Integration of nonlinear regression, curve shift, isobologram, and combination index analyses. *Clinical Cancer Research*.

[30] Ottewell PD, Mönkkönen H, Jones M, Lefley DV, Coleman RE, Holen I (2008). Antitumor effects of doxorubicin followed by zoledronic acid in a mouse model of breast cancer. *Journal of the National Cancer Institute*.

[31] Xia L, Chen D, Han R, Fang Q, Waxman S, Jing Y (2005). Boswellic acid acetate induces apoptosis through caspase-mediated pathways in myeloid leukemia cells. *Molecular Cancer Therapeutics*.

[32] Syrovets T, Büchele B, Krauss C, Laumonnier Y, Simmet T (2005). Acetyl-boswellic acids inhibit lipopolysaccharide-mediated TNF-*α* induction in monocytes by direct interaction with I*κ*B kinases. *Journal of Immunology*.

[33] Utaisincharoen P, Tangthawornchaikul N, Ubol S, Chaisuriya P, Sirisinha S (2000). Tnf-*α* induces caspase 3 (CPP 32) dependent apoptosis in human cholangiocarcinoma cell line. *Southeast Asian Journal of Tropical Medicine and Public Health*.

[34] Locksley RM, Killeen N, Lenardo MJ (2001). The TNF and TNF receptor superfamilies: Integrating mammalian biology. *Cell*.

[35] Aggarwal BB (2003). Signalling pathways of the TNF superfamily: a double-edged sword. *Nature Reviews Immunology*.

[36] Gaur U, Aggarwal BB (2003). Regulation of proliferation, survival and apoptosis by members of the TNF superfamily. *Biochemical Pharmacology*.

[37] Zamorano J, Mora AL, Boothby M, Keegan AD (2001). NF-kappaB activation plays an important role in the IL-4-induced protection from apoptosis. *International Immunology*.

[38] Takada Y, Ichikawa H, Badmaev V, Aggarwal BB (2006). Acetyl-11-keto-*β*-boswellic acid potentiates apoptosis, inhibits invasion, and abolishes osteoclastogenesis by suppressing NF-*κ*B and NF-*κ*B-regulated gene expression. *The Journal of Immunology*.

[39] Sferra R, Vetuschi A, Catitti V (2012). *Boswellia serrata* and Salvia miltiorrhiza extracts reduce DMN-induced hepatic fibrosis in mice by TGF-beta1 downregulation. *European Review for Medical and Pharmacological Sciences*.

[40] Tulubas F, Gurel A, Oran M, Topcu B, Caglar V, Uygur E The protective effects of *ω*-3 fatty acids on doxorubicin-induced hepatotoxicity and nephrotoxicity in rats.

[41] Carvalho C, Santos RX, Cardoso S (2009). Doxorubicin: the good, the bad and the ugly effect. *Current Medicinal Chemistry*.

[42] Kalender Y, Yel M, Kalender S (2005). Doxorubicin hepatotoxicity and hepatic free radical metabolism in rats: the effects of vitamin E and catechin. *Toxicology*.

[43] Kaplan M, Mutlu EA, Benson M, Fields JZ, Banan A, Keshavarzian A (2007). Use of herbal preparations in the treatment of oxidant-mediated inflammatory disorders. *Complementary Therapies in Medicine*.

[44] Ali EN, Mansour SZ (2011). Boswellic acids extract attenuates pulmonary fibrosis induced by bleomycin and oxidative stress from gamma irradiation in rats. *Chinese Medicine*.

[45] Hartmann RM, Martins MIM, Tieppo J, Fillmann HS, Marroni NP (2012). Effect of *Boswellia serrata* on antioxidant status in an experimental model of colitis rats induced by acetic acid. *Digestive Diseases and Sciences*.

[46] Dalton SR, Lee SML, King RN (2009). Carbon tetrachloride-induced liver damage in asialoglycoprotein receptor-deficient mice. *Biochemical Pharmacology*.

